# Short-term followup after surgical treatment of Ewing’s sarcoma

**DOI:** 10.4103/0019-5413.69308

**Published:** 2010

**Authors:** Shishir Rastogi, Ashok Kumar, Himanshu Gupta, Shah Alam Khan, Sameer Bakhshi

**Affiliations:** Department of Orthopaedics, All India Institute of Medical Sciences, Dr. B. R. A. Institute Rotary Cancer Hospital, Delhi, India; 1 Department of Medical Oncology, All India Institute of Medical Sciences, Dr. B. R. A. Institute Rotary Cancer Hospital, Delhi, India

**Keywords:** Ewing’s sarcoma, multimodality treatment, resection/reconstruction, arthrodesis

## Abstract

**Background:**

Results of surgical treatment in Indian patients of Ewing’s sarcoma managed with multimodality treatment with chemotherapy and/or radiotherapy are insufficient. We report a retrospective evaluation of a series of cases of Ewing’s sarcoma managed with chemotherapy, surgery with or without radiotherapy.

**Materials and Methods:**

54 patients of biopsy-proven Ewing’s sarcoma of the bone, except craniofacial and vertebral bones were included. The patients having recurrence or having previous treatment were excluded from the study. Local and systemic extent of the sarcoma was defined, staged, and patients were subjected to the chemotherapy, surgery, and in some cases radiotherapy. Patients were evaluated for results of surgery with respect to complications, recurrence, and metastases at 3, 6, 9, 12, 18 and 24 months of follow-up

**Results:**

Average age of patients was 15.6 years (range 7-26 years); average delay in treatment was 4.1 months (1-7 months); follow-up ranged from 2 to 5 years (median 3.1 years); 14 patients (25.9%) had pulmonary metastases at their initial presentation. Twenty-one patients (38.9%) underwent resection and intercalary reconstruction with bone grafting, fixed with locking plates. Allograft was also used in 11 of these. Sixteen patients underwent resection and reconstruction with endoprosthesis, while seven patients (13.0%) underwent resection and arthrodesis. An above-knee amputation was required in 7.4% (four patients). Mesh was used for containing the graft longitudinally in five patients (femoral and tibial intercalary reconstructions) and for soft tissue attachment in two patients (hip and shoulder endoprostheses). Two patients had deep wound infection. One patient presented 1 year later with implant failure. The disease-free survival at 2 years from the time of diagnosis was 57.5% (23 out of 40) for patients without preoperative metastases and 42.9% (6 out of 14) for those with preoperative metastases. Overall, the disease-free survival at 2 years was 53.7% (29 out of 54 patients). Overall survival rate at 2 years was 61.1% (33 out of 54 patients).

**Conclusion:**

Results of surgical treatment in this study are comparable with the current literature in spite of involvement of long bony segment and large soft tissue component. Intramedullary fibular autograft with morcellized cancellous autograft and allograft contained longitudinally in a mesh appears to be a good alternative with such large bone tumors.

## INTRODUCTION

Ewing’s sarcoma is a highly anaplastic, round-cell tumor of neuroectodermal origin. It is the second most common primary malignant bone tumor in children, although some studies have found it to be more common than osteosarcoma in some populations.^1^,^2^ Rao *et al*. reported an incidence of 19.4% for Ewing’s sarcoma among the primary bone tumors in India.^3^ With advances in combination therapy including chemotherapy and radiotherapy, the prognosis for Ewing’s sarcoma has improved considerably.^4^–^7^ Many studies have also shown local surgical treatment and radiotherapy to have superior results as compared to radiotherapy alone.^4^ While 5-year disease-free survival (DFS) before 1980 was as low as 25%, recent studies have reported a 5-year DFS of 37–60%.^4^–^7^ A 5-year DFS of 38.0% has been reported in an Indian scenario.^6^ We report a retrospective evaluation of a series of cases of Ewing’s sarcoma managed with chemotherapy, surgery with or without radiotherapy.

## MATERIALS AND METHODS

Patients with Ewing’s sarcoma operated between 2003 and 2007 were studied retrospectively. The eligibility criteria were children and adults with Ewing’s sarcoma of the bone excluding craniofacial bones, patients who had not received treatment elsewhere, and a histologically proven diagnosis. All patients were followed up for a minimum of 2 years or till death, if they died before 2 years. Out of a total of 57 patients treated, 3 were lost to followup at an early stage (less than 2 years) and were excluded. The study did not include patients managed with non-surgical treatment with chemotherapy and radiotherapy. At presentation, all patients were investigated with local radiographs and MRI, followed by a core biopsy. Histological assessment included hematoxylin and eosin-stained sections, staining for PAS, as well as assessment of MIC2 expression. Further staging included chest radiograph, non contrast CT (NCCT) chest, and Technitium-MDP bone scan. Besides, routine blood investigations were also done. All patients first underwent neoadjuvant chemotherapy using cycles of vincristine, actinomycin-D, cyclophosphamide, and doxorubicin alternating with courses of ifosfamide and etoposide.^8^ Minimum six cycles were given and clinical response was assessed. The patients then underwent either surgery alone or surgery along with radiotherapy. Patients having large tumor were subjected to initial radiotherapy to reduce the size of the local tumor before surgery. Two of such patients did not respond to radiotherapy and were subjected to limb ablation surgery. Surgery was done within 6 weeks after the last chemotherapy. A repeat local site radiograph, MRI, and bone scan were performed before surgery to restage the tumor. Surgical planning was based on the age of the patient, the site and local extent of the tumor, involvement of neurovascular bundle, and clinical response to chemotherapy. The options included resection followed by intercalary reconstruction with fibular graft or endoprosthesis insertion, arthrodesis, and amputation. Patients with purely diaphyseal Ewing’s sarcomas were subjected to intercalary reconstruction with fibular autograft and morcellized allograft. Patients having diametaphyseal tumors were treated with resection – endoprosthesis or resection – arthrodesis based on age and preferences of the patient. Patients having neurovascular involvement and skin fungation were treated with limb ablation surgery.

Postoperatively, wound was examined at 2^nd^ postoperative day with drain removal. Sutures were removed at 12–14 days. Patients with arthrodesis and with intercalary reconstruction were immobilized in an above-knee plaster of paris slab till suture removal followed by above-knee cast for initial 3–4 months and later mobilization in above-knee caliper brace till radiological union. Patients with endoprosthesis were immobilized in an above-knee/elbow slab or U-slab for 7–8 days followed by gradual mobilization and strengthening exercises. Adjuvant chemotherapy cycles were started 3–4 weeks after the surgery. A total of 48 weeks of chemotherapy was given including neoadjuvant and adjuvant chemotherapy. The patients were followed up with radiographs of local site and chest at 3, 6, 9, 12, 18, and 24 months, and a bone scan and NCCT chest at 6 months. Further bone scan and CT were ordered if indicated by clinical examination and radiography.

## RESULTS

**Figure 1:**
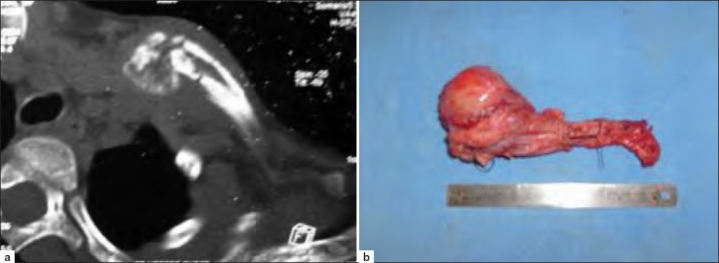
(a) Axial section of noncontrast CT scan showing Ewing’s sarcoma of the right clavicle. (b) Photograph showing the resected right clavicle with tumor *in situ*.

**Figure 2:**
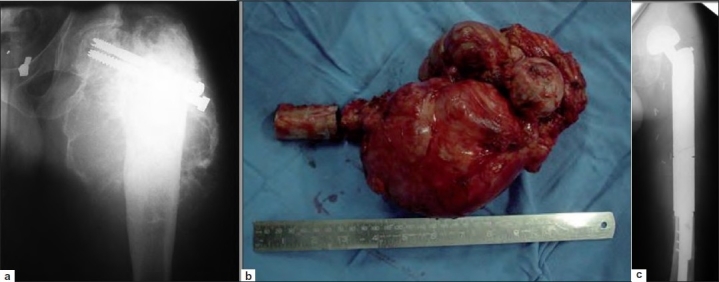
(a) Anteroposterior radiograph of left hip with thigh showing Ewing’s sarcoma with implant *in situ* (b) Photograph showing resected proximal femur with tumor *in situ*. (c) Anteroposterior radiograph of left hip with thigh showing the endoprosthesis *in situ* at 4 years of followup

**Figure 3:**
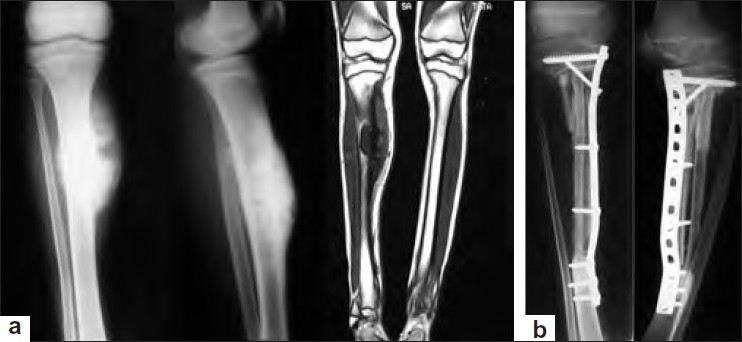
(a) Anteroposterior and lateral radiographs with T1-weighted coronal section of MRI showing Ewing’s sarcoma of proximal one-third diaphysis of right tibia. (b) Anteroposterior and lateral radiographs of right leg showing intercalary reconstruction with a well-incorporated fibular autograft of proximal one-third diaphysis of right tibia at 5-year followup

A total of 57 cases of Ewing’s sarcoma operated in this department were studied. Out of these, three patients were lost to followup soon after surgery and hence excluded. Out of the remaining 54 cases, there were 32 men and 22 women [Table 1]. Average age was 15.6 years (range 7–26 years). The average delay in presentation and treatment was 4.1 months (1-7 months). Only two patients had axially located tumors – one in clavicle [Figure 1a and b] and another in the pelvis. All other tumors were located in the tibia (10 patients), humerus (3 patients), femur (34 patients; Figure 2a–c), fibula (4 patients), and ulna (1 patient). Fourteen patients (25.9%) had metastatic disease at presentation [American Joint Committee on Cancer (AJCC) stage IVA]; all of them were having pulmonary metastases. All other patients were in AJCC stage II. Patients with stage IV tumor received 8–10 cycles of neoadjuvant chemotherapy, while others received 6 cycles of neoadjuvant chemotherapy (average 7 cycles). Twenty-two patients (40.7%) also received preoperative radiotherapy at the local site. Twenty-one of the 54 patients (38.9%) underwent resection of tumor and intercalary reconstruction fixed with locking plates with autogenous fibular graft and autogenous iliac crest corticocancellous graft in all of them [Figure 3a and b, Table 2]. This included 17 patients with femoral involvement and 4 patients with tibial involvement. Allograft was also used in 11 of these (20.4% of all patients). The tumor extended to the joint line in 23 patients. Of these, 16 patients (29.6%) underwent resection of tumor and reconstruction with endoprosthesis, while 7 patients (13.0%) underwent resection and arthrodesis. The decision was based on the age of the patient, location of tumor, as well as patient’s occupation and preference. Endoprosthesis was used in three proximal femoral tumors [Figure 2a–c], seven distal femoral, two proximal tibial, three proximal humeral, and one proximal ulnar tumors. Knee arthrodesis was performed in four patients with distal femoral and three patients with proximal tibial tumors. In arthrodesed knee, the bone gap following tumor resection was according to the length of the tumor and it was bridged with autogenous fibula along with morcellized allograft maintaining a shortening of not more than 5 cm (2-5 cm shortening). Six patients (11.2%) had only resection of the tumor in the form of partial fibulectomy (four patients), claviculectomy (one patient) [Figure 1a and b], and resection of pelvic tumor (one patient). Four patients (7.4%) had extensive tumor of the lower limb with neurovascular involvement and required an above-knee amputation (three patients of distal femur and one of proximal tibia).

**Table 1:** Patient characteristics

Average age at presentation	15.6 years
Sex	
Male	32
Female	22
Metastases at presentation	
Yes	14
No	40
Location	
Axial	2
Extremities	52

**Table 2:** Surgical procedures performed

Surgery	Number of patients	Percentage
Resection of tumor and intercalary reconstruction with autogenous fibula with allograft	11	20.4
Resection of tumor and intercalary reconstruction with autogenous fibula (without allograft)	10	18.5
Resection + endoprosthesis	16	29.6
Resection + arthrodesis	7	13.0
Fibulectomy	4	7.4
Claviculectomy	1	1.9
Pelvic resection	1	1.9
Above-knee amputation	4	7.4

In seven patients with intercalary reconstruction (*n*=5) or endoprosthesis placement (*n*=2), a polyglactin 910 or polypropylene mesh was also used. In five cases of intercalary reconstruction, the mesh was used for reattachment of muscles, tendons and capsule, and to hold the bone graft pieces. The bone gap following resection was bridged with fibular autograft surrounded by morcellized allograft. This whole reconstruction was circumferentially enclosed in mesh, and the surrounding muscles and fascia were attached to the mesh. In two cases, it was used in the periarticular area following reconstruction with endoprosthesis, for stability, and reattachment of abductors and other soft tissues.

Postoperatively, all patients with intercalary reconstruction or endoprosthesis achieved adequate functional range of motion, although all of them had varying degrees of stiffness. Out of 33 cases of lower limb Ewing’s sarcoma treated with intercalary reconstruction or endoprosthesis, twenty patients had a good functional range of motion (0–90° flexion in hip and 0–90° flexion in knee). Six patients had mild restriction of movements (5–80° flexion arc in hip and 5–80° flexion arc in knee). The remaining seven patients had a flexion deformity of 10° at hip or knee joint (range of motion 10–70° flexion at hip and 10–70° flexion at knee). Three patients with proximal humeral endoprosthesis had abduction in the range of 0–20°, flexion 0–40°, and external rotation 0–10°. One patient with elbow prosthesis had elbow range of motion 20–85°. Five patients had superficial wound complications including superficial necrosis of margins and superficial infection (one patient following fibulectomy, two patients with intercalary reconstruction, and two patients with endoprosthesis insertion). All of them improved with regular wound dressings. Two patients developed deep wound infection. Both had intercalary reconstruction with allograft placement. They required debridement of wound and removal of allograft bone chips, followed by regular wound dressings and eventual healing of wound [Table 3]. One of the patients with intercalary reconstruction of femur with fibula and long locking plate presented 1 year later with implant failure and fracture of the fibular graft. This patient required implant removal and repeat internal fixation with locking plate. Followup ranged from 2 to 5 years (median 3.1 years). Postoperatively, out of the 40 patients without preoperative metastases, 17 patients had developed local or distant recurrence by 2 year followup: 11 patients developed isolated pulmonary metastases, 5 patients developed pulmonary as well as local recurrence, and 1 patient developed isolated local recurrence. This patient with isolated local recurrence and four other patients with local recurrence underwent amputation. Three patients with one to three pulmonary nodules had pulmonary metastatectomy. In the remaining patients, the metastases were not amenable to surgical removal. Overall, 13 out of 40 patients without pre-operative metastases had died by the 2 year follow-up. Eight of the 14 patients with preoperative metastases had persistent pulmonary metastases at the time of 2-year followup or death. The disease-free survival at 2 years from the time of diagnosis was 57.5% (23 out of 40) for patients without preoperative metastases and 42.9% 6 out of 14) for those with preoperative metastases. Overall, the disease-free survival at 2 years was 53.7% (29 out of 54 patients). Overall survival rate at 2 years was 61.1% (33 out of 54 patients). Only 4 patients with pulmonary metastases were alive at final follow-up. The average delay in presentation and treatment was 4.1 months. Resection of tumor in these patients with delayed presentation required more surgical time, blood replacements, mobilization of adjascent muscles for coverage (eg. medial gastrocnemius in proximal tibia) and had relatively delayed rehabilitation.

**Table 3:** Complications and recurrence

	Number of patients
Superficial wound complications	5
Deep wound infection	2
Implant failure with fracture of fibular graft	1
Postoperative pulmonary metastases only	11
Local recurrence only	1
Local recurrence and postoperative distant metastases	5
Persistence of preoperative pulmonary metastases	8

## DISCUSSION

We evaluated retrospectively the experience of a single institution in the surgical treatment of 54 cases of Ewing’s sarcoma of bone. Although advances in multimodal therapy have considerably improved the prognosis, distant metastases still have an incidence of 37–60% at 5 years.^4^–^7^ The median time to relapse has been reported to be 2 years.^5^ Many authors have pointed out that involvement of surgical resection of tumor in the multimodal management of Ewing’s sarcoma is associated with a better outcome.^4^,^5^,^9^,^10^ Indian patients usually present late with large soft tissue component due to various reasons. This late presentation makes planning and execution of surgery difficult due to increased local vascularity, muscle involvement, and close proximity of the neurovascular bundles. Reconstruction of the residual long skeletal defect after resection of these tumors becomes more challenging due to nonavailability of the allograft. We found the disease-free survival rate in such patients following chemotherapy and surgery to be 53.7% at 2 years, which is comparable to the rates reported by other authors.^7^,^8^ While the neoadjuvant and adjuvant chemotherapy protocols were standardized in our study, the surgical part of the management was individualized based on the characteristics of the tumor as well as the patient profile. For example, a tumor extending to a joint was managed with resection and either endoprosthesis insertion or arthrodesis, depending on the age of the patient, patient’s needs and preferences, and non availability of endoprostheses. Early joint mobilization, starting at 7–8 days, in patients with endoprosthesis insertion, resulted in an acceptable functional range of motion in all patients, although varying degrees of stiffness persisted. Various modalities of intercalary and joint reconstruction using osseous allograft, osteoarticular allograft, and allograft–prosthetic composites have been described in literature.^11^–^15^ Abed *et al*. recently reported that the combined use of a vascularized fibular graft and allograft is of value as a limb-salvage procedure for intercalary reconstruction after resection of bone tumors around the knee, especially in skeletally immature patients.^16^ In our study, allograft was used in conjunction with fibular and iliac autografts in 11 patients requiring intercalary reconstruction. Two of these patients developed deep wound infection requiring debridement and removal of allograft chips. All patients achieved good functional results. In view of scarcity of allogenic bone grafts and nonavailability of bone banks in many places, intercalary reconstruction with autogenous fibula graft fixed with locking plate is a good option. Ten of our patients were offered this procedure, with good results and only minor wound complications in two of them. There was only one major complication of the reconstruction procedure due to implant pull out, which was managed with replating. None of the patients with mesh insertion developed wound discharge postoperatively and no complications which might be attributable to mesh insertion were seen. The soft tissue and fascial defects resulting from resection of tumor with wide soft tissue cuff may be covered with muscle grafts. However, in cases with extensive soft tissue dissection, the surgeon may not always have the luxury of being able to use such flaps. Often it becomes impossible to reattach the soft tissues unless the gap is covered with something. Loss of soft tissue attachments in the periarticular region also leads to instability. We have found mesh to be quite useful in all such situations. It physically bridges and stabilizes the soft tissue defect till adequate healing has occurred, and thus preserves muscle function and joint motion.

The prognosis of Ewing’s sarcoma with multimodality treatment has been proven to be better than with single modality treatment. Peng *et al*. showed an improvement in the 3-year event-free survival from a dismal 18% with single modality treatment to 46% with multimodality treatment.^17^ Addition of ifosphamide and etoposide to the chemotherapy regimens has further improved survival.^8^ In 2001, Sluga *et al*. compared their survival data for the patients of Ewing’s sarcoma presenting between 1949 to 1980 and 1981 to 1994.^7^ In the first group, only about half of the patients had surgical excision of the tumor, and all but 10.7% of the patients had radiotherapy. These figures changed to 93% patients undergoing surgery and only 51% patients receiving radiotherapy in the second group. The prognosis was significantly better in the second group. They also found a clear improvement in local tumor control by surgery. Lee *et al*. have also recently reported the absence of surgical treatment in the management of Ewing’s sarcoma to be associated with decreased overall survival in their analysis of 725 patients in a population-based cancer registry.^18^ Similar results were found by other authors, with improved rates of overall survival and recurrence-free survival in patients receiving chemotherapy and surgery with or without radiotherapy, as compared to those receiving chemotherapy and radiotherapy without surgery.^19^,^20^ The survival rates in our study are similar to the rates found in these studies using multimodality treatment including surgery.

In conclusion, with adequate neoadjuvant and adjuvant chemotherapy, surgery for local resection of tumor with individualized reconstruction procedures, and radiotherapy when required, the prognosis for the patients with Ewing’s sarcoma can be improved.
